# Endomyocardial Fibrosis Associated With Hypereosinophilic Syndrome: Diagnostic and Management Insights From a Case Report

**DOI:** 10.1002/ccd.70377

**Published:** 2025-11-26

**Authors:** Ibrahim Antoun, Benjamin Marrow, Ravi Pathmanathan, Sanjay S. Bhandari

**Affiliations:** ^1^ Department of Cardiology University Hospitals of Leicester NHS Trust Leicester UK; ^2^ Department of Cardiovascular Sciences University of Leicester Leicester UK

**Keywords:** anticoagulation, cardiac MRI, endomyocardial fibrosis, hypereosinophilic syndrome, late gadolinium enhancement, myocarditis

## Abstract

Endomyocardial fibrosis (EMF) is a rare form of restrictive cardiomyopathy associated with eosinophilic disorders, characterized by apical subendocardial fibrosis and thrombus formation. Cardiac magnetic resonance (CMR) provides a comprehensive, noninvasive evaluation, enabling diagnosis, assessment of disease activity, and guidance of therapy. A 73‐year‐old man with a history of chronic eosinophilia was referred following detection of left ventricular hypertrophy and T‐wave inversion on electrocardiogram. Transthoracic echocardiography was inconclusive. CMR revealed a nondilated left ventricle with mildly impaired systolic function, severe left atrial enlargement, and no inducible ischemia. Late gadolinium enhancement demonstrated apical subendocardial fibrosis extending into the right ventricular apex, with an overlying left ventricular thrombus. These findings were diagnostic of EMF. The patient was treated with a direct oral anticoagulant. At 8‐month follow‐up, repeat CMR showed resolution of the thrombus and persistent fibrotic scarring. Quantitative T2 mapping demonstrated normal values, excluding ongoing myocardial inflammation. Immunosuppression was therefore not initiated, and anticoagulation was continued. The patient remained clinically stable without thromboembolic events. This case highlights the pivotal role of CMR in diagnosing and managing EMF. LGE imaging provided the characteristic pattern of apical fibrosis with thrombus, while T2 mapping enabled discrimination between chronic fibrosis and active inflammation, guiding therapy away from unnecessary immunosuppression. CMR thus represents the gold‐standard imaging modality in EMF, offering both diagnostic confirmation and longitudinal monitoring of treatment response.

## 1 Introduction

Cardiovascular disease is increasing in prevalence, and its management is challenging, especially in the developing world [1, 2, 3, 4]. Endomyocardial fibrosis (EMF) is a form of restrictive cardiomyopathy characterized by fibrous thickening of the endocardium at the ventricular apex, often with calcification and thrombus formation [5]. Although classically endemic to tropical regions of Africa, Asia, and South America, nontropical cases occur, particularly in association with hypereosinophilic syndromes (HES) or Löffler endocarditis [6]. In EMF, a prodromal inflammatory phase (often subclinical) typically precedes irreversible fibrotic remodeling of the myocardium. Clinical manifestations include heart failure, arrhythmias, and systemic embolism from intracardiac thrombi [6]. Diagnosis has traditionally relied on transthoracic echocardiogram (TTE) and invasive biopsy; however, cardiac MRI (CMR) offers superior noninvasive tissue characterization. CMR can demonstrate the pathognomonic “double V” sign, a trilaminar appearance of normal myocardium, fibrotic endomyocardium, and overlying thrombus at the apex, on late gadolinium enhancement (LGE) imaging [7]. This provides not only a definitive diagnosis but also assesses disease extent, ventricular function, and thrombus burden more accurately than TTE. Although EMF is overall a rare cardiomyopathy, it remains the most prevalent cause of restrictive cardiomyopathy worldwide, particularly in endemic regions. MRI has become the cornerstone diagnostic tool in EMF because of its superior spatial resolution, tissue characterization capabilities, and ability to identify the tri‐layered apical pattern of normal myocardium, fibrotic endocardium, and thrombus. We report a case of EMF in an elderly patient with chronic eosinophilia, in whom CMR was pivotal for diagnosis and management, avoiding invasive biopsy and guiding therapy.

## 2 Case Presentation

**Figure 1:**
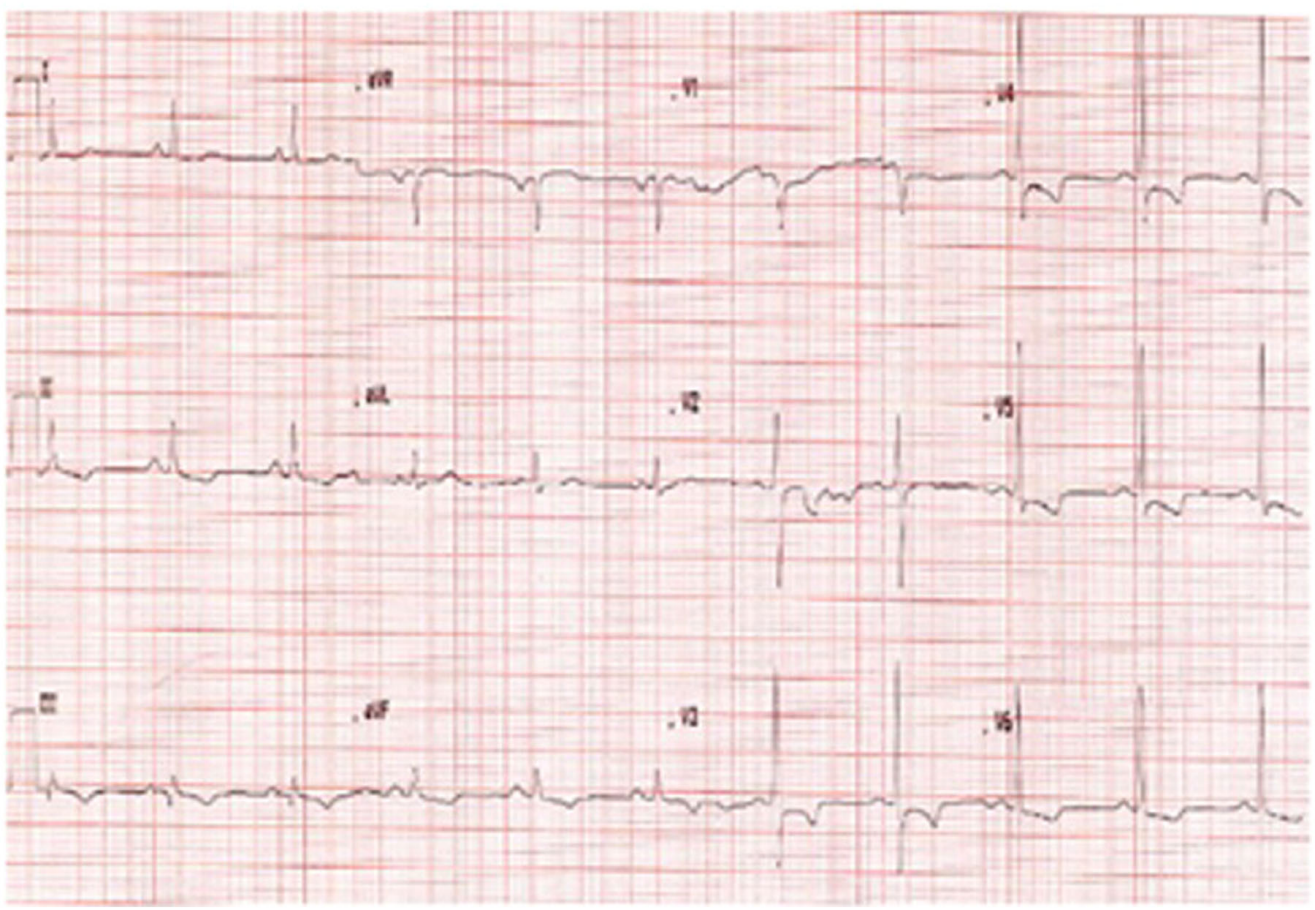
Twelve‐lead ECG showing sinus rhythm with left ventricular hypertrophy and deep T‐wave inversion in the lateral leads. These repolarization changes are typical of subendocardial myocardial disease. No acute ischemic changes were evident. (B) Long‐axis cine CMR image (four‐chamber view) demonstrating a nondilated left ventricle, a markedly dilated left atrium, and normal right ventricular size. There is mild left ventricular systolic impairment. No regional wall motion abnormality or inducible perfusion defect was seen. [Color figure can be viewed at wileyonlinelibrary.com]

A 73‐year‐old man was referred to cardiology for further evaluation after a preoperative assessment detected an abnormal ECG. He had a longstanding history of eosinophilia (absolute eosinophil counts consistently above the diagnostic threshold for HES) but no known parasitic or systemic disease. On examination, he was asymptomatic with normal vital signs. The ECG (Figure 1) showed left ventricular hypertrophy and widespread T‐wave inversion, findings consistent with those seen in EMF. TTE was unremarkable except for mild diastolic dysfunction. To exclude occult coronary disease and clarify the etiology of these changes, stress CMR was performed.

**Figure 2:**
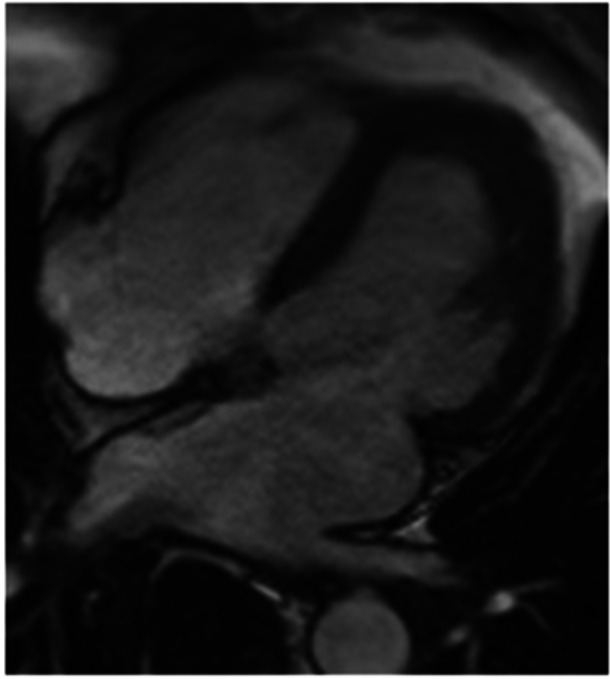
Late gadolinium enhancement CMR in the vertical long‐axis (four‐chamber) view. There is dense subendocardial enhancement confined to the apical segments of both ventricles, consistent with fibroblastic infiltration of the endocardium. LGE imaging in the short‐axis view at the apex. A well‐defined low‐signal thrombus overlies the enhanced fibrotic tissue in the LV apex. The combination of apical fibrosis (bright on LGE) with an overlying thrombus (dark) is diagnostic of endomyocardial fibrosis.

CMR cine images revealed a nondilated left ventricle with mildly reduced systolic function (LV ejection fraction ≈50%) and a severely dilated left atrium (Figure 2). There were no regional wall motion abnormalities, and inducible ischemia was absent. On LGE imaging, there was striking subendocardial enhancement confined to the apical segments of both ventricles, extending to the right ventricular apex. Overlying this enhancement was a large layered thrombus in the LV apex (Figure 2). This combination of apical subendocardial fibrosis with adherent thrombus is diagnostic of EMF. No other myocardial pathology was seen.

Given the identified thrombus, the patient was started on therapeutic anticoagulation (a direct oral anticoagulant). Since sustained eosinophilia was documented, a diagnosis of HES‐associated endomyocardial damage was made. Secondary causes of eosinophilia were excluded, and the patient was managed jointly with the hematology team. Because EMF had likely entered the chronic fibrotic phase, we deferred high‐dose steroids initially. Instead, he continued anticoagulation and standard heart failure therapy.

**Figure 3:**
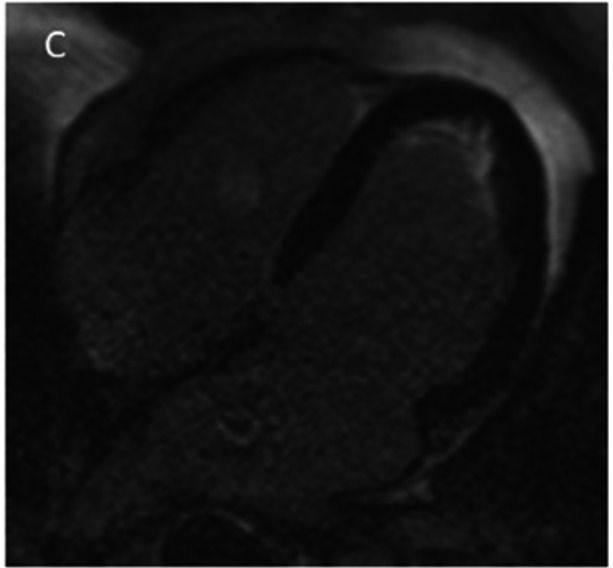
Follow‐up LGE imaging after 8 months of anticoagulation. The four‐chamber LGE image shows persistent apical endocardial enhancement, indicating stable fibrotic scarring. Importantly, the previously seen left ventricular thrombus is no longer present. The absence of a thrombus on follow‐up supports the complete resolution of the condition with anticoagulation therapy.

**Figure 4:**
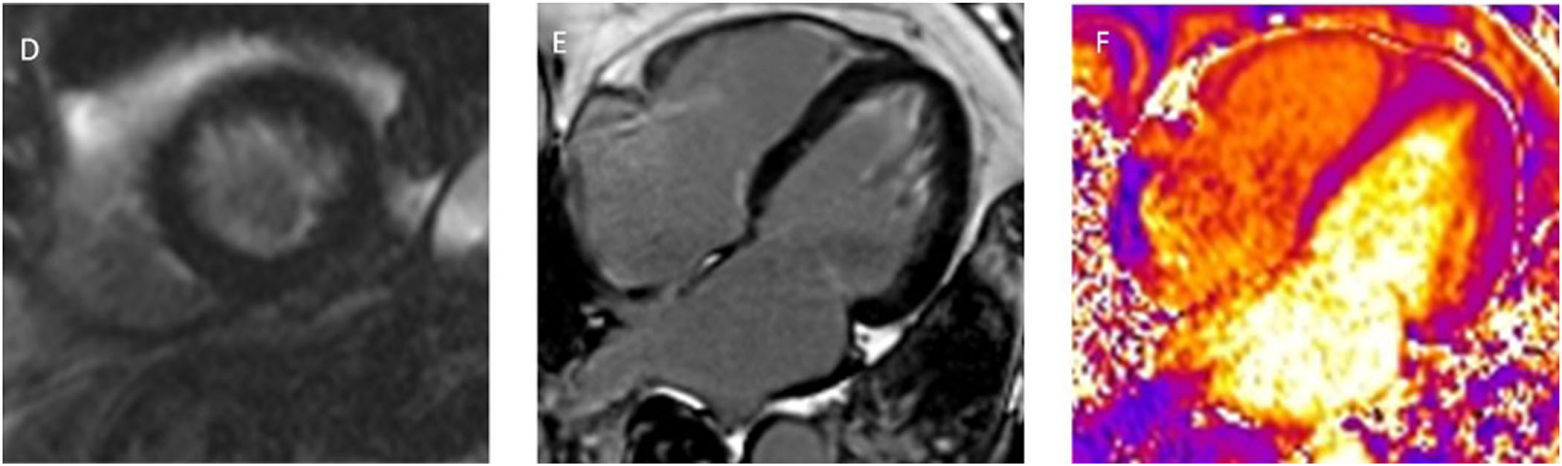
Parametric mapping summary (for illustrative purposes): (Left) Baseline imaging findings, including the pattern of apical fibrosis and thrombus on LGE, (middle) management timeline showing anticoagulation therapy, and (right) follow‐up findings with resolved thrombus and normal T2 (This schematic figure summarizes key imaging and clinical milestones of the case). [Color figure can be viewed at wileyonlinelibrary.com]

At follow‐up (8 months later), repeat CMR was obtained. The LV wall motion and function were unchanged, and all findings of EMF persisted. The apical LV thrombus had resolved completely (Figure 3). Importantly, quantitative T2 mapping was performed, revealing that all myocardial T2 relaxation times fell within the normal range (Figure 4), indicating the absence of active edema or inflammation. Considering these findings, immunosuppressive therapy was deemed unnecessary. The patient remained clinically well on continued anticoagulation and heart failure medications, with no thromboembolic events.

## 3 Discussion

This case highlights the critical role of CMR in the diagnosis and management of EMF. Initially, the patient's presentation (older age, no tropical exposure, chronic eosinophilia) was atypical for classic tropical EMF but consistent with HES‐related disease. Cardiac involvement occurs in the majority of HES cases, often beginning with an acute necrotic (myocarditic) phase and progressing to EMF [8]. Early identification of myocardial fibrosis is therefore important. CMR proved decisive, as it not only revealed the typical pattern of apical subendocardial LGE but also quantified function and identified a thrombus that was not visible on TTE [9].

The “double V” LGE pattern (normal myocardium, fibrotic endocardium, thrombus) is highly specific for EMF [10]. In our patient, LGE images clearly showed bright subendocardial enhancement in the LV apex with a hypoenhanced thrombus layer, confirming EMF without the need for biopsy. Previous studies have emphasized that CMR is more sensitive than TTE for detecting the fibrotic changes and thrombi associated with EMF [11]. In the Cleveland Clinic series of HES‐associated cardiac disease, two cases had LV thrombi seen only on CMR, missed by TTE [9]. Similarly, our patient's thrombus was identified on LGE imaging, enabling appropriate anticoagulation.

Therapeutically, EMF is challenging. In early, inflammatory stages (as in acute eosinophilic myocarditis), immunosuppression can prevent progression to fibrosis. Indeed, case reports have documented regression of EMF with high‐dose steroids and cytotoxic agents if given promptly [12]. However, once fibrosis is established, medical therapy is supportive, focusing on heart failure management and prevention of complications. Our patient's T2 map was normal, indicating no residual myocardial edema. This suggested that the active inflammatory phase had passed, so corticosteroids were unlikely to benefit. Current evidence, therefore, supports reserving immunosuppression for demonstrable active myocarditis [13]. Beyond diagnosis, MRI also provides important prognostic information. Quantification of the extent and distribution of LGE has been shown to correlate with clinical outcomes in EMF. In the cohort studied by Salemi and colleagues, the burden of LGE was associated with disease severity and adverse prognosis, supporting the use of MRI not only for defining morphology but also for risk stratification [14]. In our patient, follow‐up MRI confirmed thrombus resolution and stable fibrotic changes, providing reassurance in the absence of additional high‐risk features.

Anticoagulation is another key consideration. EMF creates a thrombogenic substrate; in HES‐related EMF, the thromboembolic risk is high. Prophylactic anticoagulation is often advised to prevent mural thrombus formation. Consistent with this, our patient's LV clot resolved with anticoagulation alone, and no embolic events occurred.

Ultimately, this case highlights the importance of CMR for follow‐up. By quantifying T2 relaxation times, we could noninvasively demonstrate the absence of active inflammation. This tissue characterization gave us confidence to avoid steroids, sparing the patient from their side effects. CMR also reconfirmed that the EMF burden was stable and that systolic function remained preserved. Regular CMR surveillance may thus allow more personalized management of EMF, guiding decisions on immunotherapy, anticoagulation, or surgical intervention if needed.

EMF should be considered in patients with unexplained eosinophilia and restrictive cardiomyopathy features. CMR is the preferred modality for identifying its hallmarks (apical fibrosis and thrombus) and for staging disease activity. In our patient, CMR led to a definitive diagnosis and informed management. Anticoagulation resolved the thrombus, and CMR tissue mapping safely ruled out ongoing myocarditis, thereby avoiding unnecessary immunosuppression.

In conclusion, the case demonstrates the pivotal role of CMR in diagnosing EMF in an elderly patient with hypereosinophilia. Characteristic LGE patterns and thrombus detection confirmed EMF noninvasively. Follow‐up CMR with T2 mapping helped tailor therapy by excluding active myocardial inflammation. Thus, CMR not only establishes the diagnosis of EMF but also guides treatment decisions and monitoring in such patients.

## Consent

The authors confirm that the patient gave informed consent to publish this case report.

## Conflicts of Interest

The authors declare no conflicts of interest.

## Data Availability

Data regarding this case report is available on request from the corresponding author.

## References

[1] I. Antoun , M. Aljabal , A. Alkhayer , et al., “Atrial Fibrillation Inpatient Management Patterns and Clinical Outcomes During the Conflict in Syria: An Observational Cohort Study,” Perfusion 40 (2024): 02676591241259140. 10.1177/02676591241259140 PMC11951463 38830625

[2] I. Antoun , A. Alkhayer , M. Aljabal , et al., “Thirty‐Day Unplanned Readmissions Following Hospitalization for Atrial Fibrillation in a Tertiary Syrian Center: A Real‐World Observational Cohort Study,” Heart Rhythm O2 5, no. 12 (2024): 854–859. 39803619 10.1016/j.hroo.2024.05.010 PMC11721721

[3] I. Antoun , A. Alkhayer , A. J. Eldin , et al., “Gender Disparity in Quality of Life in Patients With Atrial Fibrillation During the Syrian Conflict: An Observational Cohort Study,” Heart Rhythm O2 6 (2025): 362–367. 40201668 10.1016/j.hroo.2024.12.010 PMC11973665

[4] I. Antoun , A. Alkhayer , A. J. Eldin , et al., “Gender Disparity in Oral Anticoagulation Therapy in Hospitalised Patients With Atrial Fibrillation During the Ongoing Syrian Conflict: Unbalanced Treatment in Turbulent Times,” Journal of Clinical Medicine 14, no. 4 (2025): 1173. 40004703 10.3390/jcm14041173 PMC11855938

[5] A. Bondue , C. Carpentier , and F. Roufosse , “Hypereosinophilic Syndrome: Considerations for the Cardiologist,” Heart 108, no. 3 (2022): 164–171. 34172539 10.1136/heartjnl-2020-317202

[6] R. Mankad , C. Bonnichsen , and S. Mankad , “Hypereosinophilic Syndrome: Cardiac Diagnosis and Management,” Heart 102, no. 2 (2016): 100–106. 26567231 10.1136/heartjnl-2015-307959

[7] F. P. de Carvalho and C. F. Azevedo , “Comprehensive Assessment of Endomyocardial Fibrosis With Cardiac MRI: Morphology, Function, and Tissue Characterization,” Radiographics 40, no. 2 (2020): 336–353. 32004118 10.1148/rg.2020190148

[8] J. Caudron , Y. Arous , J. Fares , V. Lefebvre , and J. N. Dacher , “Endomyocardial Fibrosis in the Context of Hypereosinophilic Syndrome: The Contribution of Cardiac MRI,” Diagnostic and Interventional Imaging 93, no. 10 (2012): 790–792. 22819397 10.1016/j.diii.2012.04.019

[9] M. Reeder , Y. Okushi , S. L. P. Vega , et al., “The Cleveland Clinic Experience of Eosinophilic Myocarditis in the Setting of Hypereosinophilic Syndrome: Demographics, Cardiac Imaging, and Outcomes,” Cardiovascular Diagnosis and Therapy 14, no. 6 (2024): 1122–1133. 39790190 10.21037/cdt-24-347 PMC11707478

[10] I. S. Syed , M. W. Martinez , D.‐L. Feng , and J. F. Glockner , “Cardiac Magnetic Resonance Imaging of Eosinophilic Endomyocardial Disease,” International Journal of Cardiology 126, no. 3 (2008): e50–e52. 17399811 10.1016/j.ijcard.2007.01.019

[11] J. H. Butterfield , G. C. Kane , and C. R. Weiler , “Hypereosinophilic Syndrome: Endomyocardial Biopsy Versus Echocardiography to Diagnose Cardiac Involvement,” Postgraduate Medicine 129, no. 5 (2017): 517–523. 28440714 10.1080/00325481.2017.1317215

[12] A. D. Klion , “How I Treat Hypereosinophilic Syndromes,” Blood 126, no. 9 (2015): 1069–1077. 25964669 10.1182/blood-2014-11-551614 PMC4551360

[13] M. Brambatti , M. V. Matassini , E. D. Adler , K. Klingel , P. G. Camici , and E. Ammirati , “Eosinophilic Myocarditis,” Journal of the American College of Cardiology 70, no. 19 (2017): 2363–2375. 29096807 10.1016/j.jacc.2017.09.023

[14] V. M. Salemi , C. E. Rochitte , A. A. Shiozaki , et al., “Late Gadolinium Enhancement Magnetic Resonance Imaging in the Diagnosis and Prognosis of Endomyocardial Fibrosis Patients,” Circulation. Cardiovascular imaging 4, no. 3 (2011): 304–311. 21415124 10.1161/CIRCIMAGING.110.950675

